# Antinociceptive effect of chrysin in diabetic neuropathy and formalin-induced pain models

**DOI:** 10.1080/19768354.2020.1765019

**Published:** 2020-05-25

**Authors:** Jae-Seung Hong, Jing-Hui Feng, Jung-Seok Park, Hee-Jung Lee, Jae-Yong Lee, Soon-Sung Lim, Hong-Won Suh

**Affiliations:** a Department of Physical Education, College of Natural Science, Hallym University, Chuncheon, Korea; b Institute of Natural Medicine, Department of Pharmacology, College of Medicine, Hallym University, Chuncheon, Korea; c Department of Biochemistry, College of Medicine, Hallym University, Chuncheon, Korea; d Department of Food Sciences and Nutrition, College of Natural, Health, and Life Sciences, Hallym University, Chuncheon, Korea

**Keywords:** Chrysin, antinociception, opioid receptors, spinal CREB protein

## Abstract

Chrysin, a natural flavonoid, is the main ingredient of many medicinal plants, which shows potent pharmacological properties. In the present study, the antinociceptive effects of chrysin were examined in ICR mice. Chrysin orally administered at the doses of from 10 to 100 mg/kg exerted the reductions of formalin-induced pain behaviors observed during the second phase in the formalin test in a dose-dependent manner. In addition, the antinociceptive effect of chrysin was further characterized in streptozotocin-induced diabetic neuropathy model. Oral administration chrysin caused reversals of decreased pain threshold observed in diabetic-induced peripheral neuropathy model. Intraperitoneally (i.p.) pretreatment with naloxone (a classic opioid receptor antagonist), but not yohimbine (an antagonist of α2-adrenergic receptors) or methysergide (an antagonist of serotonergic receptors), effectively reversed chrysin-induced antinociceptive effect in the formalin test. Moreover, chrysin caused a reduction of formalin-induced up-regulated spinal p-CREB level, which was also reversed by i.t. pretreated naloxone. Finally, chrysin also suppressed the increase of the spinal p-CREB level induced by diabetic neuropathy. Our results suggest that chrysin shows an antinociceptive property in formalin-induced pain and diabetic neuropathy models. In addition, spinal opioid receptors and CREB protein appear to mediate chrysin-induced antinociception in the formalin-induced pain model.

## Introduction

Chrysin (5,7-dihydroxyflavone) is contained in numerous plants and propolis (Mani and Natesan 2018). Chrysin has been widely used as a supplement for health benefits (Samarghandian et al. 2017). However, recent studies have reported that chrysin also shows potent pharmacological properties such as anti-inflammatory, immunoregulatory, antioxidant, anticancer, and antiviral activities (Pushpavalli et al. 2010; Sun et al. 2012; Ahad et al. 2014; Song et al. 2015; Zeinali et al. 2017). A recent study has reported that propolis or Dysphania gaveolens, which contain chrysin as one of components exerts an antinociceptive effect in the writhing and hot-plate tests (Déciga-Campos et al. 2017; Sun et al. 2018). Furthermore, a recent study has demonstrated that chrysin was effective in reducing the nociception in formalin pain test (Farkhondeh et al. 2015). However, an earlier study has demonstrated that chrysin does not show the antinociceptive effect (Mada et al. 2009). Furthermore, chrysin produces hyperalgesia (Zhai et al. 2008), revealing that the exact role of chrysin in the regulation of nociception is still remained controversial.

cAMP response element binding protein (CREB) modulates various types of pain transmission. For example, CREB protein expressions are altered in dorsal root ganglia (DRG) and the spinal cord following nociceptive pain. The study (Crown et al. 2006) implicated CREB proteins in the allodynia development and spinal cord injury animal model. In addition, CREB protein expression is found to contribute to several pain models, such as neuropathic pain model, diabetic neuropathy model, capsaicin-treated pain model (Miyabe and Miletic 2005; Song et al. 2005; Wu et al. 2005; Dang et al. 2014). However, the possible role of spinal CREB protein in the regulation of chrysin-induced antinociception is not clear yet.

Several lines of evidence have demonstrated that chrysin possesses an anti-inflammatory effect (Mada et al. 2009; Ha et al. 2010). Among numerous pain models, the formalin-induced pain is regarded as an inflammation-induced pain (Hunskaar and Hole 1987). In addition, several studies have reported that one of the major pathological causes of diabetic neuropathy is inflammation. Although chrysin was used variety of pain tests, the present study was designed to examine the possible antinociceptive effect of chrysin mainly in inflammation-induced pain models such as formalin-induced pain and diabetic-induced neuropathy models. Furthermore, we tried to reveal the possible molecular mechanisms in chrysin-induced antinociception.

## Materials and methods

### Experimental animals

These experiments were approved by the University of Hallym Animal Care and Use Committee (Registration Number: Hallym R2014-60). All procedures were conducted in accordance with the ‘Guide for Care and Use of Laboratory Animals’ published by the National Institutes of Health and the ethical guidelines of the International Association for the Study of Pain.

Male ICR mice (MJ Co., Seoul, Korea) weighing 20–25 g were used for all the experiments. Animals were housed 5 per cage in a room maintained at 22 ± 0.5°C with an alternating 12 h light–dark cycle. Food and water were available *ad libitum*. The animals were allowed to adapt to the laboratory for at least 2 h before testing and were only used once. Experiments were performed during the light phase of the cycle (10:00–17:00).

### Production of streptozotocin-induced diabetic neuropathy model

Diabetic neuropathy animal model was produced using streptozotocin administration. 0.1 mg/kg of streptozotocin was administered intraperitoneally (i.p.) once. The experiment was performed in 5 weeks after streptozotocin injection.

### Von-Frey test

Antinociception and Mechanical allodynia were assessed by Von-Frey tests (Bonin et al. 2014). For the measurement of the Von-Frey test, mice were individually placed in a clear glass cell with a metal mesh floor allowed to adapt to the testing environment for 30 min, and then Von-Frey filaments (North Coast Medical, Inc., Gilroy, CA, USA) were applied to the plantar surface using an up and down paradigm.

### Intraplantar formalin test

For the formalin test (Hunskaar et al. 1985), 10 μl of 5% formalin was injected subcutaneously under the plantar surface of the left hind paw. Following the injection of formalin, the animals were immediately placed in an acrylic observation chamber, and the time spent licking, shaking and biting the injected paw was measured with a stop-watch timer and considered as indication of nociception. The early phase of the nociceptive response normally peaked 0–5 min, and the last phase 20–40 min after formalin injection, representing the direct effect on nociceptors and inflammatory nociceptive responses, respectively (Hunskaar and Hole 1987). Animals were orally pretreated with chrysin (from 10 to 80 mg/kg) with 30 min prior to performing the formalin test.

### Pretreatment of antagonists

At first, mice were pretreated i.p. with either saline, naloxone (5 mg/kg), methysergide (5 mg/kg), or yohimbine (5 mg/kg), 10 min before oral administration of vehicle as a control or a fixed dose of chrysin (100 mg/kg). And then, the formalin-induced nociceptive behavior was observed.

### Protein extraction and western blot

The lumbar section of the spinal cord of mice was dissected. Tissue was washed two times with cold Tris-buffered saline (20 mmol/L Trizma base and 137 mmol/L NaCl, pH 7.5). Immediately after washing, tissue was lysed with sodium dodecyl sulfate lysis buffer (62.5 mmol/L Trizma base, 2% w/v sodium dodecyl sulfate, 10% glycerol) containing 0.1 mmol/L Na_3_VO_4_, 3 mg/mL aprotinin, and 20mmolL NaF. After brief sonication, the concentration of protein was determined with a detergent-compatible protein assay reagent (Bio-Rad Laboratories, Hercules, CA, USA) using bovine serum albumin as the standard. After adding bromophenol blue (0.1% w/v), the proteins were boiled, separated by electrophoresis in 6–10% polyacrylamide gels, and transferred onto a polyvinylidene difluoride membrane (Millipore, Bedford, MA, USA). The membranes were immunoblotted with antibodies p-CREB (Abcam, 1:1000) and β-actin (Cell Signaling Technology, 1:1000) in a blocking buffer for overnight. Membranes were then washed 4 times with Tris-buffered saline containing 20% Tween-20 (TBST; 10 mM Trizma base, pH8.0, 150 mM NaCl, and 20% Tween 20) for 20 min and then incubated with the anti-rabbit IgG-horseradish peroxidase conjugate (1:4000) in blocking buffer at room temperature for 1 h. After washing the membranes with TBST for 20 min (4 times), ECL-plus solution (Millipore, Billerica, MA, USA) was added. The membranes were then exposed to a Luminescent Image Analyzer (LAS-4000, Fuji Film Co., Japan) for the detection of light emission. The specific signals were quantified with the Multi-Gauge Version 3.1 (Fuji Film, Japan) and expressed as the percentage of the control.

### Drugs

Chrysin, streptozotocin, naloxone, yohimbine and methysergide were purchased from Sigma-Aldrich (St. Louis, MO, USA). All drugs were prepared just before use.

### Statistical analysis

Statistical analysis was assessed by one-way ANOVA with Bonferroni’s post-hoc test using GraphPad Prism Version 4.0 for Windows (GraphPad Software, San Diego, CA, USA). *P*-values less than 0.05 were considered to indicate statistical significance. All values were expressed as the mean ± S.E.M.

## Results

### Effect of chrysin on pain behavior in the formalin test

**Figure 1.**
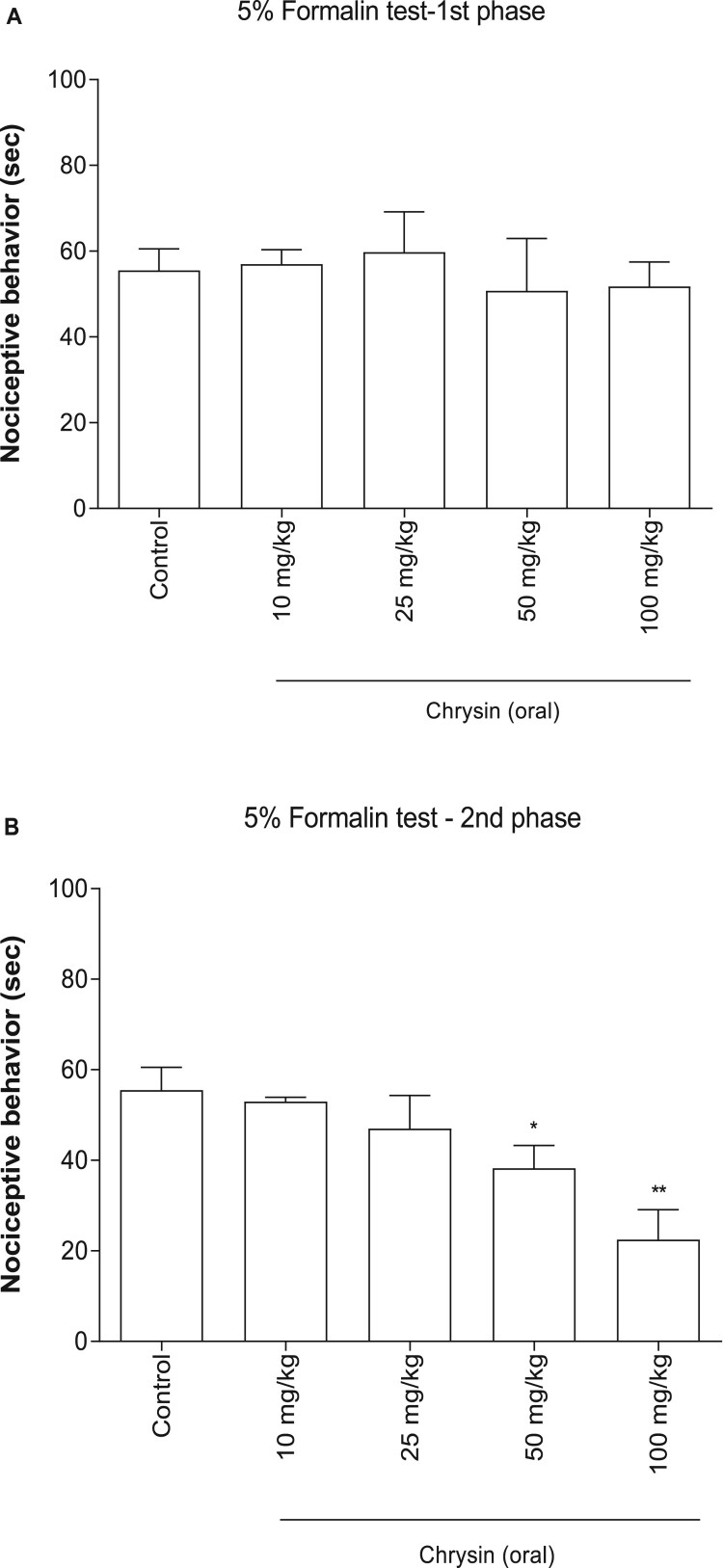
Effect of chrysin on the nociceptive response induced by formalin. Mice were administered orally with chrysin (from 10 to 100 mg/kg) for 30 min prior to the formalin (5%, 10 μl) injection subcutaneously into the plantar aspect of the left side hind paw. The cumulative response time of licking, biting and shaking the injected paw was measured during the period of 0–5 min (1st phase) (A) and 20–40 min (2nd phase) (B). The vertical bars indicate the standard error of the mean. The number of animals used for each group was 8–10 (*p < 0.05; **p < 0.01, compared with control group).

As shown in Figure 1, in the vehicle-treated control group, intraplantar injection of 5% formalin caused acute nociceptive formalin responses, which lasted for 5 min (first phase response), and then lead to chronic inflammatory response, which began about 20 min to 40 min after formalin administration (second phase response). Oral administration of chrysin (from 10 to 100 mg/kg) attenuated the nociceptive behaviors induced by formalin injection as compared with the control group during the only second phase (Figure 1).

### Effect of chrysin on pain behavior in diabetic-induced neuropathy model

**Figure 2.**
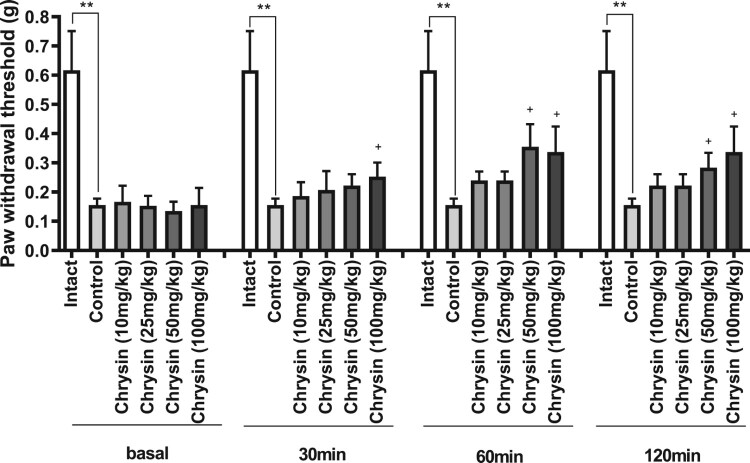
Antinociceptive effect of chrysin in diabetic neuropathy model. Diabetic neuropathy animal model was produced by injecting i.p. with 0.1 mg/kg of streptozotocin once. The experiment was performed in 5 weeks after streptozotocin injection. The vertical bars indicate the standard error of the mean. The number of animals used for each group was 8–10 (**p < 0.01, compared with intact group; ^+^*p* < 0.05, compared with control group).

As revealed in Figure 2, at 5 weeks after i.p. administration once with streptozotocin (Furman 2015), the threshold of mechanical stimulation was reduced as manifested by Von-frey filament test. Various doses (from 10 to 100 mg/kg) of chrysin were orally administered 30 min prior to the mechanical pain test. The mechanical pain threshold was measured using von-frey filament. As shown in Figure 2, chrysin caused a reversal of decreased pain threshold in a dose-dependent manner.

### Changes of phosphorylated CREB protein in the spinal cord by chrysin in the formalin-induced pain and the diabetic-induced neuropathy models

**Figure 3.**
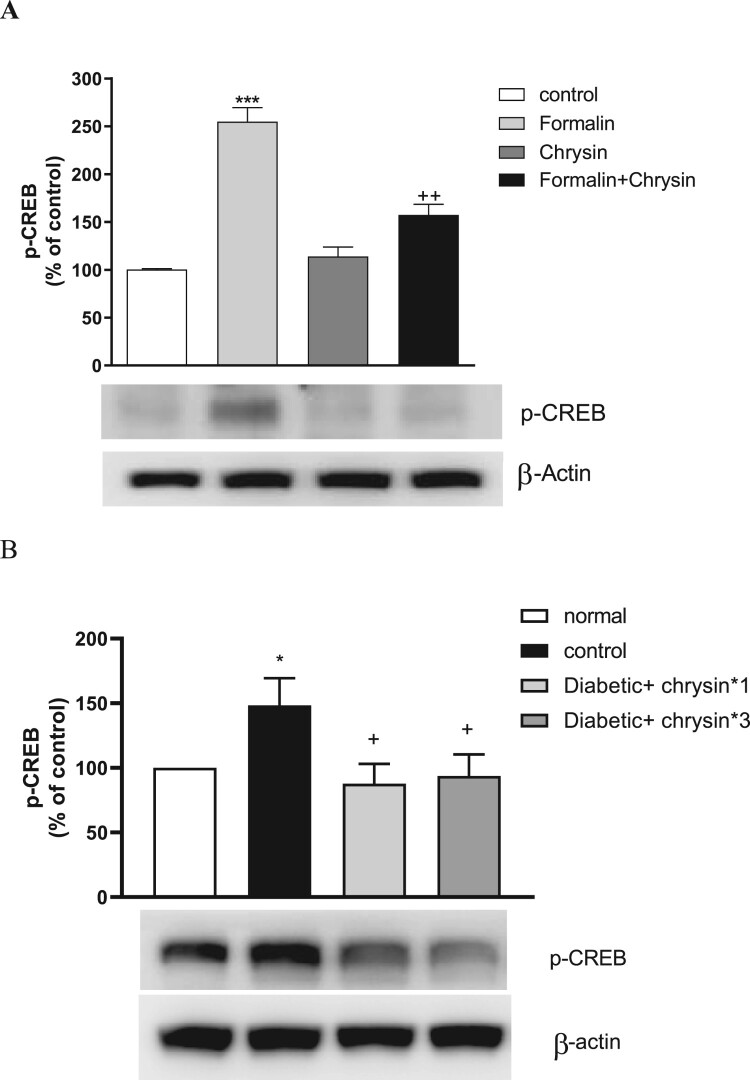
Changes of phosphorylated CREB protein in the spinal cord by chrysin in the formalin-induced pain and diabete-induced nueropathy pain models. A. The proteins were extracted from dissected lumbar spinal cord 30 min after formalin injection for Western blot analysis. The number of animals in each group is 6. B. The experiment was performed at 5 weeks after streptozotocin injection. The diabetic mice were treated for chrysin for once or 3 times. The 3 times treatment of chrysin was performed twice each day (10 am and 4 pm). The proteins were extracted from dissected lumbar spinal cord 1hr after chrysin oral administration for Western blot analysis. β-Actin (1:1000 dilution) was used as an internal loading control. Signals were quantified with the use of laser scanning densitometry and expressed as a percentage of the control. Values are mean ± SEM (***P < 0.001, compared to Control group; ^++^*P *< 0.01, compared to formalin-treated group; *P < 0.05, compared to Normal group; ^+^*P *< 0.05, compared to Diabetic group).

To assess if the expression of CREB protein phosphorylation is altered in the spinal cord after formalin injection, the proteins were extracted from dissected lumbar spinal cord at 30 min after formalin injection. As shown in Figure 3A, formalin injection caused up-regulations of p-CREB expression in the spinal cord. In addition, oral administration with chrysin (100 mg/kg) attenuated formalin-induced p-CREB level (Figure 3A). We further examined the change of p-CREB expression level in the spinal cord in streptozotocin-induced neuropathy model. In the diabetic model, phosphorylation of CREB was significantly increased compared to normal mice. However, this increase was suppressed by the treatment of 100 mg/kg chrysin either once or 3 times (Figure 3B).

### Effect of naloxone, yohimbine or methysergide on pain behavior in the formalin test

**Figure 4.**
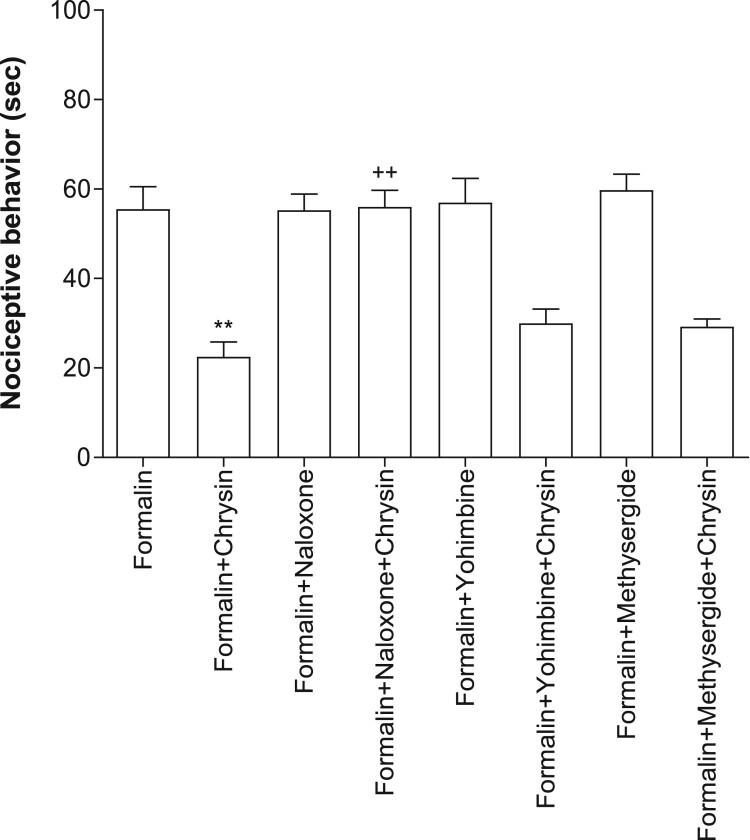
Effect of naloxone, yohimbine or methysergide on antinociception induced by chrysin in the formalin test. After i.p. pretreatment with naltrexone, yohimbine or methysergide (5 mg/kg) for 10 min, mice were administered orally with chrysin (100 mg/kg) for 30 min prior to the formalin (5%, 10 μl) injection subcutaneously into the plantar aspect of the left side hind-paw. The number of animals in each group is 6. The vertical bars indicate the standard error of the mean. (**p < 0.01, compared with formalin-treated group; ^++^*p* < 0.01, compared with formalin+chrysin group).

We next examined the possible involvement of opioidergic, serotonergic and adrenergic system in chrysin-induced antinociception. As shown in Figure 4, the blockade of opioid receptor with i.p. pre-administration of naloxone (5 mg/kg) caused a reversal of chrysin-induced antinociceptive effect. However, i.p. pretreatment with methysergide (serotonergic receptor antagonist; 5 mg/kg) or yohimbine (α-2 adrenergic receptor blocker; 5 mg/kg) did not affect chrysin-induced antinociception. The treatment of naloxone, methysergide or yohimbine itself did not affect the writhing response (Figure 4).

### Effect of naloxone on chrysin-induced down-regulation of spinal p-CREB expression in the formalin test

**Figure 5.**
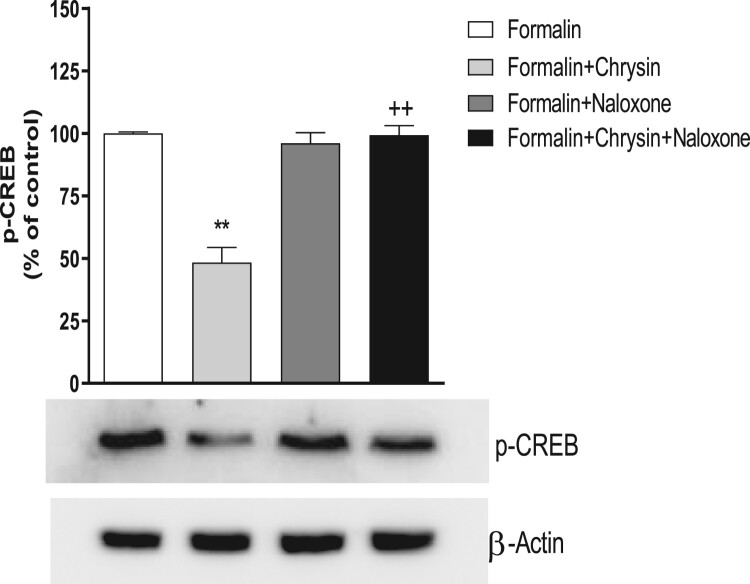
Effect of naloxone on antinociception spinal p-CREB expression induced by chrysin in the formalin test. After i.p. pretreatment with naloxone (5 mg/kg) for 10 min, mice were administered orally with chrysin (100 mg/kg) for 30 min prior to the formalin (5%, 10 μl) injection subcutaneously into the plantar aspect of the left side hind-paw. The proteins were extracted from dissected lumbar spinal cord 30 min after formalin injection for Western blot analysis. The number of animals in each group is 6. β-Actin (1:1000 dilution) was used as an internal loading control. The vertical bars indicate the standard error of the mean. (**p < 0.05, compared with formalin group; ^++^*p* < 0.05, compared with formalin+chrysin group).

Since naloxone causes a reversal of chrysin-induced antinociceptive effect in the formalin test, the present study was also designed to examine if naloxone causes the reversal of reduction of spinal p-CREB expression by chrysin. As shown in Figure 5, i.p. pretreatment with naloxone (5 mg/kg), which did not alter the basal p-CREB expression, caused a reversal of chrysin-induced down-regulation of p-CREB expression.

## Discussion

We observed in the present study that chrysin exerts an antinociceptive effect in formalin-induced pain model. Oral administration with chrysin reduced the pain behavior observed during the 2nd phase, but not the first phase in the formalin test. It has been well characterized that the intraplantar injection produces a biphasic reaction (Barrot 2012). The pain behavior observed during the first phase is mostly due to the direct stimulation of nociceptors, whereas the pain behaviors observed during the second phase involves both inflammatory mechanisms and central sensitization within the dorsal horn, suggesting that pain behaviors observed during the first and second phases are differentially regulated (Tjølsen et al. 1992). Our results indicated that chrysin is mostly effective in relieving the inflammation-induced pain behavior induced by formalin injection into the plantar of the hind-paw of mice. However, our result is contrasted with one previous study by Farkhondeh et al. (2015) in that the 150 mg/kg chrysin decreased formalin-induced pain during the first phase. In their experiments, multiple doses of chrysin were pre-treated intraperitoneally (i.p.) 60 min before formalin injection in rats. Thus, we further in the present study found that chrysin even at high dose (200 mg/kg) was not effective to exert an antinociceptive effect on the first phase in the formalin model (data not shown). According to these pieces of evidence, it is speculated that these differential effects of chrysin may be resulted from the differential origins of animal, species, or the dose and route of drug administration.

Previously, we examined the antinociceptive effect of anti-inflammatory drugs, such as aspirin and acetaminophen, when the potency was compared, chrysin showed a better potency compared to that of aspirin and acetaminophen. In our previous study, aspirin at doses of 100 mg/kg or acetaminophen at doses of 200 mg/kg significantly attenuated pain behavior induced by formalin in the second phase (Kwon et al. 2005). However, in the present study, chrysin reduced antinociception in formalin-induced inflammatory models only at doses of 50 mg/kg. Based on these results, chrysin may be applied as an effective antinociceptive drug.

To examine if chrysin is also effective in modulating mechanically-induced pain, we have produced diabetic neuropathy model. Five weeks after the production of streptozotocin-induced diabetic neuropathy model, we examined the possible antinociceptive effect of chrysin and found that chrysin is also effective in relieving diabetic-induced neuropathy. Additionally, we observed that treatment of chrysin did not alter the blood glucose level of the diabetic mice, indicating that the treatment of chrysin resulted in an antinociceptive effect, but not related to the anti-diabetic effect. Similarly to our results, Wojnar et al. (2020) suggested that chrysin reveals antioxidative activity in the lenses but shows no antihyperglycemic or antiglycation properties.

CREB protein is closely associated with pain transmission. For example, p-CREB expression in the spinal cord or dorsal root ganglia are up-regulated in various types of chronic pain models, such as neuropathic pain and neuropathy (Miyabe and Miletic 2005; Song et al. 2005). Besides, p-CREB expression in the spinal cord or brain regions are elevated in an acute inflammatory pain model such as formalin pain model (Hermanson and Blomqvist 1997; Seo et al. 2008; Hagiwara et al. 2009; Mao et al. 2013; Tochiki et al. 2015). We found in the present study that the induction of formalin pain resulted in a significant increase in the level of p-CREB expression in the spinal cord. In addition, chrysin attenuated formalin-induced up-regulation of spinal p-CREB expression. Furthermore, we observed that treatment of chrysin suppressed up-regulation of spinal p-CREB expression in the diabetic neuropathy model. These results are likely suggested, in part, that the reduction of nociception by chrysin appears to be mediated as a result of the reduction of p-CREB level in the spinal cord both in the formalin-induced pain and diabetic neuropathy models.

The opioid, serotonergic and adrenergic receptors are implicated in the modulation of nociceptive processing. For example, opioid receptors are contributed to the antinociception (Yaksh 1979; Schmauss and Yaksh 1984; Yaksh 1984). Also, the blockade of the spinal serotonergic receptors by spinal injection of methysergide, as well as the spinal noradrenergic receptors by yohimbine, antagonize the antinociception induced by morphine administered supraspinally (Yaksh 1979; Jensen and Yaksh 1984; Wigdor and Wilcox 1987). We observed in the present study that opioid receptors, but not α-2 adrenergic and serotonergic receptors, appear to be involved in orally administered chrysin-induced antinociception. In addition, we found in the present study that spinal naloxone pretreatment causes the reversal of chrysin-induced antinociception in the formalin test. Our results suggest that the reduction of nociception by chrysin treatment in formalin pain model appears to be mediated, at least, by the opioid receptors and p-CREB protein in the spinal cord. Although direct study was not performed, the results of the present study suggest that chrysin may cause the release of endogenous opioid into the spinal cord, in turn, resulting in the blockade of the formalin-induced pain transmission in the spinal cord level. It is further speculated that endogenous opioid released in to the spinal cord by chrysin appears to stimulate opioid receptor, causing chrysin-induced down-regulation of p-CREB level in the spinal cord in the formalin test.

In conclusion, our results suggest that chrysin shows an antinociceptive property in the formalin-induced pain and diabetic neuropathy models. Furthermore, this antinociceptive effect induced by chrysin may be mediated by opioid receptor, but not α-2 adrenergic receptor and serotonergic receptors. Furthermore, spinally located CREB protein might be also involved in chrysin-induced antinociception.
